# Surface-Modified Gold Nanoparticles with Folic Acid as Optical Probes for Cellular Imaging

**DOI:** 10.3390/s8106660

**Published:** 2008-10-24

**Authors:** Shiao-Wen Tsai, Jiunn-Woei Liaw, Fu-Yin Hsu, Yi-Yun Chen, Mei-Jhih Lyu, Ming-His Yeh

**Affiliations:** 1 Institute of Biochemical and Biomedical Engineering, Chang Gung University 259 Wen-Hwa 1st Rd., Kwei-Shan, Tao-Yuan, 333, Taiwan; 2 Department of Mechanical Engineering, Chang Gung University, 259 Wen-Hwa 1st Rd., Kwei-Shan, Tao-Yuan, 333, Taiwan. E-Mail: markliaw@mail.cgu.edu.tw; 3 Institute of Bioscience and Biotechnology, National Taiwan Ocean University, Taiwan Keelung, Taiwan; 4 FEOL Module Department / Platform 2 Division R&D; Taiwan Semiconductor Manufacturing Company, Ltd. E-Mail: msyehb@tsmc.com

**Keywords:** Gold nanoparticles, folic acid, laser scanning confocal microscopy, ligand-receptor endocytosis, surface plasmon resonance

## Abstract

In this study, we demonstrate that the uptake rate of the surface-modified gold nanoparticles (GNPs) with folic acid by specific cells can be increased significantly, if the membranes of these cells have sufficient folic-acid receptors. Two human breast cancer cell lines were studied; one is MDA-MB-435S cell, and the other T-47D cell. The expression of the folic acid receptors of the former is much higher than that of the latter. These cells were incubated with media containing bare GNPs or GNPs conjugated with folic acid individually. Due to the unique optical behavior (i.e. surface plasmon resonance) of GNPs, the uptake amount of GNPs by cells can be identified by using the laser scanning confocal microscopy. Our experiments show that the uptake amount of GNPs in MDA- MB-435S cells is higher than that in T-47D cells for the same culture time, if the culture medium contains bare GNPs. Moreover, if the GNPs conjugated with folic acid are used for the culture, the uptake rate of GNPs by MDA-MB-435S cells is improved more. In contrast, the uptake rates of both GNPs are almost the same by T-47D cells. The phenomenon indicates that the uptake rate of GNPs can be improved via the ligand- receptor endocytosis, compared with the nonspecific endocytosis. Therefore, the uptake rate of GNPs conjugated with folic acid by MDA-MB-435S cells is higher than that of bare GNPs.

## 1. Introduction

Stem cells, regardless of their origin, are a promising source of cells for tissue and cell engineering studies. Stem cell research relies on identifying the mechanism that underlies the differentiation of these cells and their expression of unique proteins. Therefore, understanding the protein functionality is extremely important for conducting proteomics and genomics research. Moreover, localizing or labeling proteins with small and specific probes without interfering with protein functionality is important for studying cellular fate. Most cells in living organism are approximately 10 μm in diameter; however, the components of these cells are much smaller and are in the submicron range. Most proteins, in particular, are typically less than 10 nm in size. Over the past decades, numerous dye molecules with different excitation and emission spectra have been developed for labeling cells. By combining different kinds of fluorophores and fluorescent proteins for cell staining and by irradiating a given sample of interest with different-wavelength lasers by using a laser scanning confocal microscope (LSCM), a multifluorescence image can be obtained. However, the problem of crosstalk is sometimes encountered in multifluorescence imaging due to the overlap of the emission spectra of different dyes.

Therefore, in order to interpret the signals of different biomarkers, it is important to distinguish between the emission spectra of the biomarkers by spectral unmixing of the signal acquisition data. In addition, due to the photobleaching of dye molecules, fluorophores are not suitable for long-term observation studies. Quantum dots (QDs) [1, 2] and metallic nanoparticles (MNPs) [3, 4] have been developed as cellular tracers for long-term studies. QDs offer the advantage of a narrow emission spectrum, high levels of expression, and long-term use. However, the use of QDs does not eliminate the problem of crosstalk since the mechanism of light emission from QDs is the same as that underlying light emission from fluorescence dye molecules. Further, Hsieh *et al.* [5, 6] demonstrated that the labeling of human bone mesenchymal stem cells with QD prevented their differentiation. Compared with QDs, MNPs such as gold nanoparticles (GNPs) [7-9], gold nanorods [10, 11], nanoshells [12], and nanocages [13], are more promising biomarkers that have been recently developed. Experiments have demonstrated that MNPs exhibit good biocompatibility [14] and nontoxicity [15, 16]. Moreover, the unique optical response of surface plasmon resonance (SPR) of MNPs was studied intensively. During the last decade, interdisciplinary efforts have been expended to evaluate the size and shape of various nanoparticles and to characterize the various properties of nanostructured materials. The optical properties of metallic particles ranging from microclusters to nanoparticles have been investigated mainly with respect to size. The unique optical response of spherical GNPs analyzed by SPR spectroscopy is characterized by a single absorption band that can be attributed to collective dipole oscillation. Due to the SPR of MNPs, which is a collective oscillation of electrons in the metal, a strong light scattering from MNPs can be induced when MNPs are irradiated by a light within range from ultraviolet (UV) to near-infrared (NIR). Therefore, a single-wavelength continuous-wave laser is used for MNP excitation, the light scattered by the MNPs is monochromatic and has a wavelength that is identical to that of the laser.

In addition to possessing a tunable SPR response and serving as a useful biomaterial, gold is an inert metal well-known for its biocompatibility. Gold surfaces possess considerable potential as simple substrates for the self-assembly of antibodies or various other biological substances. The internalization of nanoparticles into specific cells is a critical step in the labeling of cellular components. Nanoparticles are usually internalized by fluid-phase endocytosis, receptor-mediated endocytosis, or phagocytosis. The extent of internalization of metallic nanoparticles is severely limited by the low efficiency of uptake of these particles by endocytosis. Therefore, in order to increase the uptake of nanoparticles by target cells, the nanoparticle surface is modified with a ligand known to be efficiently internalized by target cells via receptor-mediated endocytosis. Surface functionalizing GNPs by conjugation with a specific antibody, e.g., anti-epidermal growth factor receptor (EGFR) [17] for epithelial cancer cells, has been developed for the application of these particles in the diagnosis and thermo-phototherapy of cancer cells [18]. Darkfield imaging [19, 20] has also been used to demonstrate the expression of scattered light from GNPs in cell membranes but not from GNPs in the cytoplasm. Although some studies have reported the use of GNPs for cellular imaging [10-13], a systematical study comparing GNPs with fluorophores as biomarkers for cellular imaging by LSCM is yet to be carried out.

The objectives of this study are as follows: (1) to improve the uptake of GNPs by employing a surface-modifying agent such as folic acid (FA) [21] and (2) to investigate the optical response of GNPs by using LSCM for the application of GNPs in cellular imaging. For these purposes, two human breast cancer cell lines are studied; one is the human mammary carcinoma cell lines (MDA-MB-435S, HTB-129), and the other the human breast ductal carcinoma cell lines (T-47D, BCRC 60250). The expression of the FA receptors on the membranes of MDA-MB-435S cells is more significant than that of T-47D cells [22]. Therefore, we adopt these two cell lines for comparison to study the efficiency of receptor-mediated endocytosis of GNPs conjugated with FA. The nuclei of these cells were stained with propidium iodide (PI). The scattering expression of GNPs and the fluorescence of PI can be observed simultaneously by LSCM [23]. Since the intensity of the scattered light is proportional to the amount of GNPs, the LSCM cellular images can be used to qualitatively determine the amount of GNPs. The images show that GNPs are distributed in the cytoplasm. In addition, the uptake rate of GNPs conjugated with FA is higher than that of bare GNPs. In addition, transmission electron microscope (TEM) images demonstrated that the GNPs are aggregated within cytoplasmic vesicles and are not attached to the cell membrane. This phenomenon illustrates that the receptor-mediated endocytosis enhances the internalization of GNPs by target cells. This study proposes a rapid method for preparing biopsy specimens, which involve labeling these specimens with GNPs modified with specific molecule instead of employing traditional labeling methods used in biomedical applications.

## 2. Materials and Methods

### 2.1 Synthesis of GNPs

In our experiment, GNPs were synthesized by using the seed-growth method. The size of the GNPs (30 nm in diameter) was controlled by tuning the seed-growth process. Ascorbic acid, cetyltrimethylammonium bromide (CTAB), and NaBH_4_ were purchased from ACROS. The other chemicals were of reagent grade and were purchased from SIGMA. The GNPs were prepared by the method of Jana *et al.* [24], which involves using a surfactant-based seed-mediated sequential growth system. Briefly, 20 ml of citrate-stabilized GNPs (seed crystals) were prepared by reducing a 20 mL solution containing HAuCl_4_ and citric acid (both of the same concentration) with ice cold aqueous 0.1 M NaBH_4_ (0.6 mL). An aqueous growth solution (9 mL) containing CTAB (0.08 M), HAuCl4 (0.25 mM), acetone (3 mL) and cyclohexane (4 mL) was used. The growth solution and seed crystal were mixed together and maintained at room temperature for a minimum of 4 h.

### 2.2 Surface modification of GNPs with FA

Monolayers of organosulfur adsorbates on metal surfaces are generally prepared by vapor deposition [25], chemisorption [26], or electrochemical deposition [27]. Metalloorganic materials can be more conveniently prepared by using a thiol-stabilized colloidal gold solution [28, 29]. This involves immersing a metal surface into a saturated solution containing thiol, removing it, washing it with a small amount of the same solvent, and then drying it. Briefly, the GNPs were dispersed in 0.25 mM 2-mercaptoethylamine in ethanol, and the mixture was maintained at room temperature for 1 h. This mixture was then centrifuged; the resulting pellet was washed several times with dd H_2_O. The precipitate was resuspended in an aqueous solution containing a mixture of FA and *N*-(3-dimethyl-aminopropyl)-*N′*-ethylcarbodiimide hydrochloride (EDAC); the molar ratio of FA to EDAC in this solution was 10:1. The pH of the solution was adjusted to 4.75. The solution was then incubated at room temperature with continuous stirring. After 4 h, the pH of the solution was adjusted to 9 in order to stop the reaction. The solution was then centrifuged and the precipitate was collected.

### 2.3 Cell culture

Human breast cancer cells (MDA-MB-435S) and human breast ductal carcinoma cells (T-47D) were used in the experiments. MDA-MB-435S cells were grown in Dulbecco's Modified Eagle Medium (DMEM), and T-47D cells in RPMI-1640 medium supplemented with 10% fetal bovine serum (FBS). These cultures were placed in a humidified atmosphere of 5% CO_2_ at 37°C. The media were replaced once every three days. The cells were cultured to confluence on slides in a 10-mm petri dish. The cells were then incubated with a medium containing two types of GNPs; one is the surface- modified GNP with FA (GNP-FA) and the other is bare GNP. The latter was used as the control. In order to study the efficiency of the intracellular uptake of GNPs-FA and bare GNPs by these two cell lines, these cells were incubated with these nanoparticles for different time periods (e.g. for 5 min and 30 min). After the incubation at 37°C, the cells were fixed with 4% paraformaldehyde solution for 20 min at room temperature, and then treated with RNase solution for 20 min to remove the cytoplasmic RNA. Subsequently, these cells were incubated with PI solution for 3 min to stain the DNA in the nuclei of these cells. At last, these samples were washed for several times for the following measurement of LSCM.

### 2.4 Characterization

UV/VIS/Near-IR spectrometry: In order to ensure that there is no alteration in the SPR features of the GNPs after the surface modification of FA, the SPR spectra of different solutions containing bare GNPs, GNPs conjugated with 2-mercaptoethylamine, and GNPs-FA were measured using a UV/VIS/Near-IR spectrometer (Perkin Elmer Lambda 900).

Fourier Transform Infrared (FT-IR) and Nuclear Magnetic Resonance (NMR) Spectra: The Fourier transform infrared FT-IR spectra of bare GNPs, GNPs conjugated with 2-mercaptoethylamine, and GNPs-FA were measured by using an FT-IR spectrometer (HORIBA, FT-730). To verify that the 2- mercaptoethylamine and FA have been immobilized onto the surface of GNPs, NMR spectra were also measured by using a Mercury 400 NMR (400 MHz). All samples were milled with potassium bromide (KBr), and a pellet of the mixture was pressed into a disc for analysis. D_2_O was used as the solvent for all samples.

Laser scanning confocal microscope (LSCM): By using lasers, strong light scattering from the GNPs and fluorescence from PI could be induced simultaneously. The GNP and PI signals could be detected separately by using 2 bandpass filters. Compound LSCM images are obtained without cross-talk. The lasers and filters used for the LSCM analysis are the same as those used in our previous study [23]. The peak of the SPR band of aggregated GNP is at 540 nm, and the excitation band of PI is from 450 nm to 600 nm; the maximum excitation occurs at 535 nm. The emission band of PI ranges from 585 nm to 720 nm; the maximum emission of fluorescence is at 620 nm. Thus, in this study, a diode- pumped solid-state (DPSS) laser of 561 nm is used for LSCM imaging, and two corresponding bandpass filters are adjusted to separate the scattered light and the fluorescence emission; filter-I (508– 572 nm) is used for the detection of the scattered light from GNPs, and filter-II (604–668 nm) for the fluorescence emission from PI.

Transmission electron microscope (TEM): In order to understand the distribution/location of nanoparticles in the cells, the cells were incubated with the GNPs and GNPs-FA for an additional 24 h after being grown to confluence on a petri dish. The cells were detached, centrifuged, and the cell pellet obtained was washed with phosphate-buffered saline (PBS). Subsequently, the pellet was fixed with 2.5% glutaraldehyde (GA) in PBS for 30 min. The pellet was then washed with PBS several times and then postfixed in 1% osmium tetroxide in PBS for 1 h. After several rinses with PBS, the pellet was dehydrated by suspending it in an ethanol solution. After dehydration, ultrathin, Spurr-embedded sections of the pellet were stained with uranyl acetate and lead citrate. TEM images were recorded using a JEOL 1230 electron microscope.

## 3. Results and Discussion

**Figure 1.**
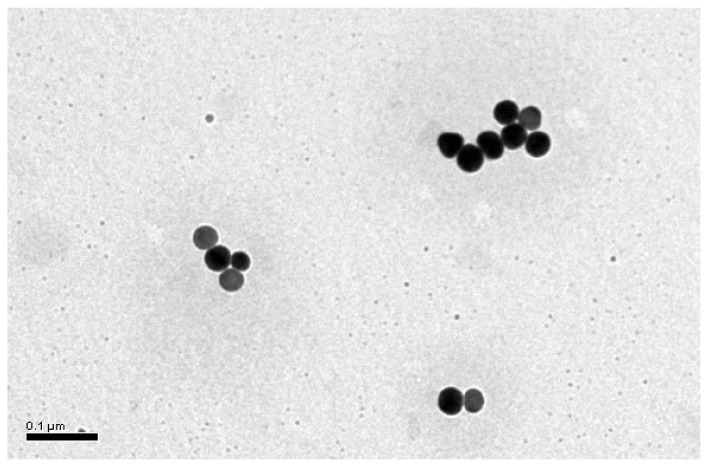
Transmission electron microscopy image of GNPs at a magnification of 100,000, where the scalebar denotes 100 nm. The average diameter of the GNPs is 27.9±9.1nm.

GNPs were synthesized by using the wet chemical method as described in the previous section. The average diameter of these GNPs is 27.9±9.1 nm, which is determined by selecting 30 GNPs randomly from a TEM photograph as shown in Figure 1.

**Figure 2.**
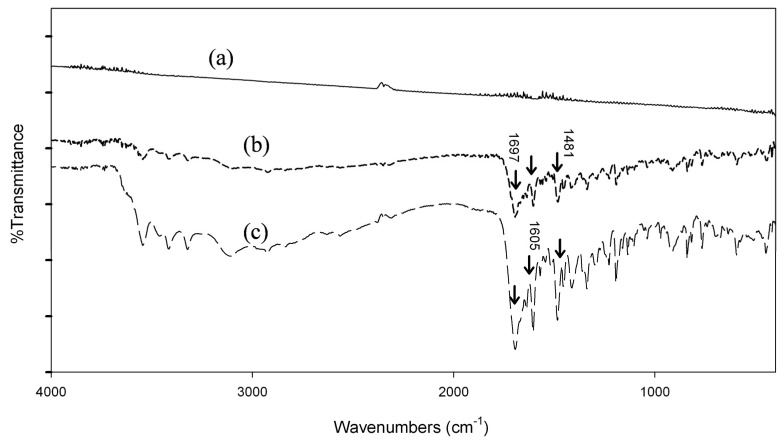
FTIR spectra of (a) naked GNPs, (b) surface-modified GNPs with 2-mercapto- ethylamine and folic acid, and (c) pure folic acid.

Figure 2 shows the FT-IR spectra of bare GNPs, FA, and GNPs-FA. Comparing these three spectral lines, the spectrum of GNPs-FA exhibits the characteristic IR absorption peaks of FA at 1605 cm^−1^ (benzene, conjugated double absorption), 1697 cm^−1^ (ester bond), and 1481cm^−1^ (hetero-ring, conjugated double bond).

**Figure 3.**
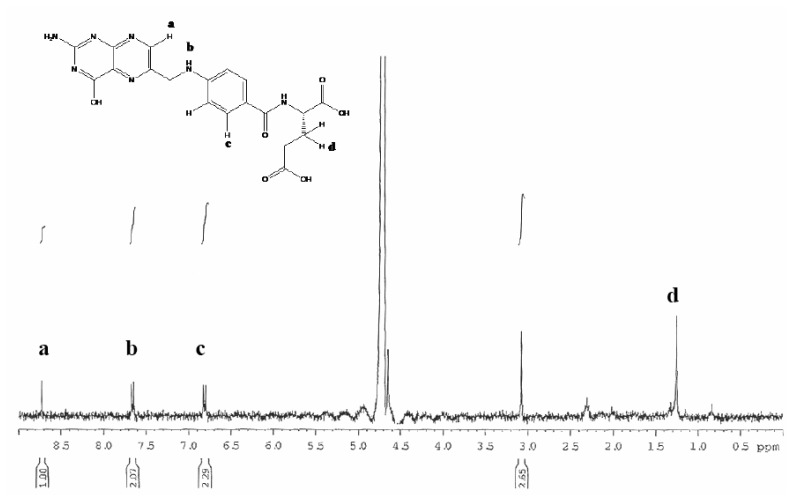
NMR spectrum of the surface-modified GNPs with folic acid; the structure of folic acid is shown in the left top.

**Figure 4.**
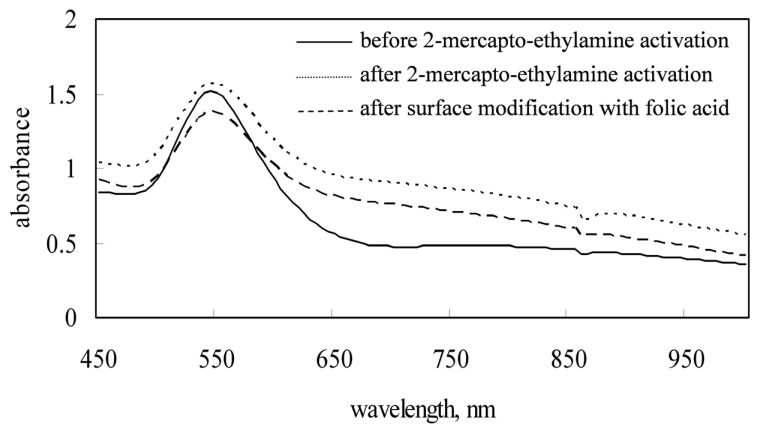
Surface plasma resonance spectra of GNPs before and after the surface modification with folic acid.

The NMR spectrum of these GNPs-FA was further measured to demonstrate that FA has been immobilized onto the surface of GNPs (as shown in Figure 3), according to ^1^H-NMR chemical shift: δ 8.73 (H_A_, s, 1H), δ 7.66 (H_C_, d, *J*=8.2Hz, 2H), and δ 6.81 (H_E_, d, *J*=8.2Hz, 2H). Using the UV-VIS- NIR spectrophotometer, the absorption spectra of different aqueous solutions containing bare GNPs, GNPs conjugated with 2-mercaptoethylamine, and GNPs-FA were measured, as depicted in Figure 4. Figure 4 shows that the specific SPR adsorption peak of the GNPs is not altered by the chemical surface modification of folic acid.

Since the expression of FA receptors on the membranes of MDA-MB-435S cells is more significant than that of T-47D cells [22], we adopted these two cell lines for comparison to study the efficiency of receptor-mediated endocytosis of GNPs-FA. In addition, we set up two bandpass filters of different ranges for LSCM. With the aid of these two filters, two cellular images are acquired separately. Subsequently, a compound image is obtained by merging these 2 images, because the ranges of the two filters do not overlap. The wavelength range of filter-I is adjusted, such that only the scattered light from GNPs is detected but not the fluorescence of PI. On the other hand, the wavelength range of filter-II is adjusted such that only the fluorescence of PI is detected but not the scattered light. Moreover, using a pinhole, the out-of-focus signals are excluded so that only the optical section image of the focal plane is obtained. Combining the images of filter-I and filter-II, the compound image of the expressions of GNP and PI can be obtained. The size of the confocal pinhole is adjusted to be 1.12 and 1.3 Airy units for PI and the GNPs, respectively. If there are some GNPs are internalized by cells, some lightspots resulting from the light scattering of GNPs can be observed by LSCM. Each lightspots corresponds to a cluster of GNPs in a vesicle. Therefore, via LSCM image, the uptake amount of GNPs and GNPs-FA by MDA-MB-435S and T-47D cells can be evaluated roughly by counting the number of lightspots.

In order to ensure no signal contamination from cell's autofluorescence, two control groups of MDA-MB-435 and T-47D cells were tested without the culture of GNPs in advance by using the same conditions; one group is not stained with PI, and the other group is stained with PI. In the case of the first control group, there is no signal detected. In contrast, for the second control group, only the fluorescence emission of PI from the nuclei is observed (not shown here). Actually, by altering the focal plane, the optical-section LSCM images at different depths can be obtained.

**Figure 5.**
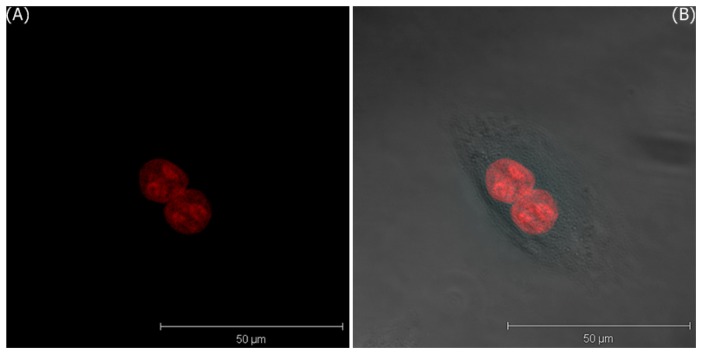
The 3D projection images of MDA-MB-435S cells incubated with bare GNPs in media after 5 minutes; A) LSCM image, and B) the merged image of LSCM and the phase contrast images.

**Figure 6.**
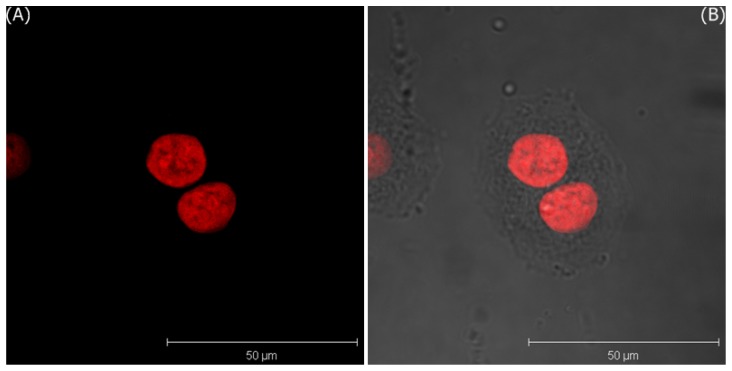
The 3D projection images of T-47D cells incubated with bare GNPs in media after 1 hour; A) LSCM image, and B) the merged image of LSCM and the phase contrast images.

Figure 5(A) only shows the 3D projection LSCM image of MDA-MB-435S cells treated with bare GNPs for 5 minutes and irradiated by DPSS laser of 561 nm, and Figure 5(B) shows the merged image of LSCM and the phase contrast images. These images are visualized by two pseudo-colors according to the ranges of filter-I (green: 508–572 nm) and filter-II (red: 604–668 nm). In these images, the red area represents the expression of PI in the nuclei. The images of the T-47D cells incubated with bare GNPs for one hour are shown in Figure 6.

**Figure 7.**
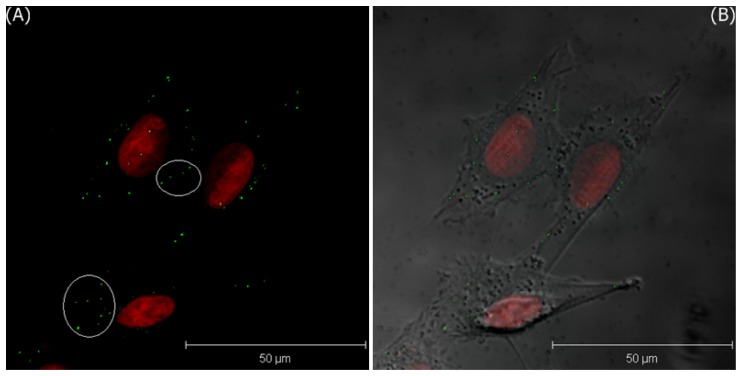
The 3D projection images of MDA-MB-435S cells incubated with bare GNPs in media after 30 minutes; A) LSCM image, and B) the merged image of LSCM and the phase contrast images, where the green lightspots are the expression of GNPs.

According to the conclusion of our previous study [23], if GNPs are internalized by these cells, some lightspots will be observed in the cytoplasm, due to the light scattering from the cluster of GNPs in the cellular vesicles. However, no expression of green lightspots is observed in both cells. It is attributed that the culture time with bare GNPs is too short for the nonspecific endocytosis. If the incubation time is increased to 30 min, several green lightspots are observed within the cytoplasm of MDA-MB-435S cells to indicate that some GNPs are internalized, as shown in Figure 7.

**Figure 8.**
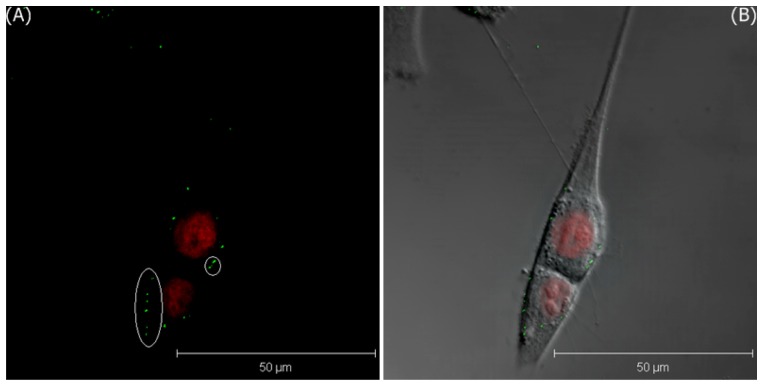
The 3D projection images of MDA-MB-435S cells incubated with GNPs-FA in media after 5 minutes; A) LSCM image, and B) the merged image of LSCM and the phase contrast images, where the green lightspots are the expression of GNPs.

**Figure 9.**
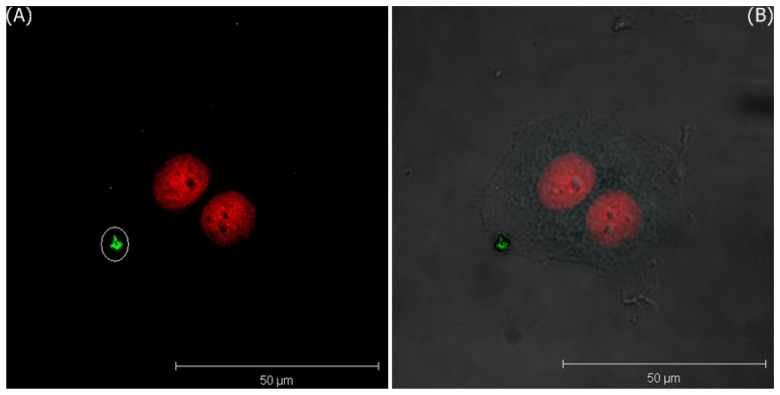
The 3D projection images of T-47D cells incubated with GNPs-FA in media after 5 minutes; A) LSCM image, and B) the merged image of LSCM and the phase contrast images, where the green lightspots are the expression of GNPs.

Although these cells could uptake these GNPs via the nonspecific endocytosis, the uptake rate is still lower than that of the receptor-mediated endocytosis. To study the uptake rate of the FA-receptor endocytosis, the two cell lines were further treated with GNPs-FA for only 5 min. Figures 8 and 9 show the images of LSCM of MDA-MB-435S cells and T-47D cells, respectively. In Figure 8, a few of green lightspots inside the cells are found to indicate that certain of GNPs are endocytosed by the MDA-MB-435S cells. In contrast, the expression of GNPs is only found on the membranes of T-47D cells, but not in the cytoplasm. This is to say that FA-receptor endocytosis can promote the uptake rate of GNPs-FA for MDA-MB-435S cells, but not for T-47D cells, because the expression of FA-receptor of MDA-MB-435S cells is much stronger than that of T-47D cells.

Disease progression and viral mutation are known to occur at a very fast rate. However, most traditional diagnostic or detection methods yield results only after several hours or days. In order to obtain rapid results, the duration of detection or diagnosis should be as short as possible. The use of GNPs as biomarkers for cellular imaging enables the analysis time to be reduced.

Our previous study [23] shows that GNPs can be internalized by cytoplasmic vesicles via nonspecific endocytosis. Cells can uptake GNPs through endocytosis or receptor-mediated endocytosis; however, the efficiency of these two modes of endocytosis is very different. Target cells uptake more nanoparticles through nonspecific endocytosis when cultured for long periods and/or with a high concentration of nanoparticles (data not shown here). Surface modification of GNPs with a specific molecule (ligand) not only reduces the time of internalization of the nanoparticles by the cells but also excludes most signals resulting from nonspecific endocytosis.

In our study, FA is covalently bonded to the surface of GNPs, so this bonding does not alter the SPR characteristics of GNPs. Since the expression of FA receptors of T-47D cells is lower than that of MDA-MB-435S cells, the uptake rate of GNPs-FA by MDA-MB-435S cells is significantly faster than that of T-47D cells. The LSCM images demonstrated that ligand-receptor endocytosis could enhance the rate of uptake of GNPs-FA after a short incubation period. Using this method, we are able to label cells with a fluorescence dye and with the surface-modified GNPs with specific ligands, e.g. folic acid, within a very shot time to speed up the inspection. Furthermore, we propose the method of LSCM to facilitate the measurement of GNPs. The quantitative measurement of GNPs is still conducted.

## 4. Conclusions

In this study, we demonstrated that the uptake rate of GNPs-FA by specific cells (e.g. MDA-MB-435S cells) can be improved significantly, if the membranes of these cells have sufficient folic-acid receptors. In addition, we developed a technique to separate the scattered light of GNPs and the fluorescence of dye PI by using two bandpass filters for LSCM imaging. In LSCM image, the uptake amount of GNPs by the cell can be evaluated roughly but quickly by counting the number of lightspots in cytoplasm; each lightspot is the expression of light scattering generated from a vesicle enclosing a cluster of GNPs [23]. Since GNP can be surface functionalized by any specific bioconjugation, it will be a targeted ligand-linking biomarker [30] for specific inspection or therapy.
